# *CACNA1E* Variants Affect Beta Cell Function in Patients with Newly Diagnosed Type 2 Diabetes. The Verona Newly Diagnosed Type 2 Diabetes Study (VNDS) 3

**DOI:** 10.1371/journal.pone.0032755

**Published:** 2012-03-09

**Authors:** Maddalena Trombetta, Sara Bonetti, MariaLinda Boselli, Fabiola Turrini, Giovanni Malerba, Elisabetta Trabetti, PierFranco Pignatti, Enzo Bonora, Riccardo C. Bonadonna

**Affiliations:** 1 Department of Medicine, University of Verona, Verona, Italy; 2 Azienda Ospedaliera Universitaria Integrata di Verona, Verona, Italy; 3 Department of Life and Reproduction Sciences, University of Verona, Verona, Italy; Universita Magna-Graecia di Catanzaro, Italy

## Abstract

**Background:**

Genetic variability of the major subunit (*CACNA1E*) of the voltage-dependent Ca^2+^ channel Ca_V_2.3 is associated to risk of type 2 diabetes, insulin resistance and impaired insulin secretion in nondiabetic subjects. The aim of the study was to test whether *CACNA1E* common variability affects beta cell function and/or insulin sensitivity in patients with newly diagnosed type 2 diabetes.

**Methodology/Principal Findings:**

In 595 GAD-negative, drug naïve patients (mean±SD; age: 58.5±10.2 yrs; BMI: 29.9±5 kg/m^2^, HbA1c: 7.0±1.3) with newly diagnosed type 2 diabetes we: 1. genotyped 10 tag SNPs in *CACNA1E* region reportedly covering ∼93% of *CACNA1E* common variability: rs558994, rs679931, rs2184945, rs10797728, rs3905011, rs12071300, rs175338, rs3753737, rs2253388 and rs4652679; 2. assessed clinical phenotypes, insulin sensitivity by the euglycemic insulin clamp and beta cell function by state-of-art modelling of glucose/C-peptide curves during OGTT. Five *CACNA1E* tag SNPs (rs10797728, rs175338, rs2184945, rs3905011 and rs4652679) were associated with specific aspects of beta cell function (p<0.05−0.01). Both major alleles of rs2184945 and rs3905011 were each (p<0.01 and p<0.005, respectively) associated to reduced proportional control with a demonstrable additive effect (p<0.005). In contrast, only the major allele of rs2253388 was related weakly to more severe insulin resistance (p<0.05).

**Conclusions/Significance:**

In patients with newly diagnosed type 2 diabetes *CACNA1E* common variability is strongly associated to beta cell function. Genotyping *CACNA1E* might be of help to infer the beta cell functional phenotype and to select a personalized treatment.

## Introduction

Ca_V_2.3 is one non–L-type high-voltage-activated (HVA) channel belonging to the voltage-gated Ca^2+^ channel family, whose members mediate Ca^2+^ entry into cells in response to membrane depolarization [1].

Ca_V_2.3, which is expressed primarily in neuronal cells [2] and in pancreatic beta cells [1], plays a key role in second-phase insulin release [3]. Silencing Ca_V_2.3 expression in INS-1 cells blunts glucose-stimulated insulin secretion [4]. Ca_V_2.3^−/−^ mice display fasting hyperglycemia and impaired glucose tolerance [5], [6].

Ca_V_2.3 is composed of a major pore-forming alpha 1 E subunit (*CACNA1E*), which determines most of the channel's properties, and of multiple auxiliary subunits (alpha 2, beta, delta, gamma) [1].

Two earlier studies, performed in two different ethnic groups, reported that *CACNA1E* variants are associated to type 2 diabetes risk [7], [8]. However, their findings differed because in one study the metabolic phenotype associated to *CACNA1E* variability was insulin secretion [8], in the other study it was insulin sensitivity [7]. Both studies used insulin concentrations after a secretory stimulus to explore beta cell function. However, insulin levels are determined by both beta cell function and insulin clearance, which is in turn influenced by insulin sensitivity. Thus, any insulin based assessment of beta cell function also reflects insulin clearance. Indeed, in the former study [8], the association between *CACNA1E* variability and beta cell function was sought using either surrogate indexes of glucose-induced insulin secretion during OGTTs, or insulin levels in the late part of IVGTTs. In the latter study [7], only acute insulin response after i.v. glucose challenge was measured, i.e. a facet of beta cell function in which Ca_V_2.3 should play a nonsignificant role [3]. Finally, in both studies, associations between *CACNA1E* variability and metabolic phenotypes were investigated in nondiabetic individuals [7], [8]. We hypothesized that, if common *CACNA1E* variability were relevant in determining beta cell function, this might be evident also when overt diabetes ensues and such an information would be of potential clinical interest.

We therefore undertook the present study to test whether the genetic variability of *CACNA1E* affects key metabolic phenotypes (beta cell function and insulin sensitivity, both assessed by state-of-art methods) in patients with newly diagnosed type 2 diabetes.

The *CACNA1E* region was tested by genotyping a number of tag SNPs, selected to capture most (∼93%) of common *CACNA1E* variability. We used the database of the Verona Newly Diagnosed Type 2 Diabetes Study (VNDS), a cohort in which associations between common genetic variants and metabolic phenotypes have already been shown to be detectable [9], [10], possibly because of the absence of the potentially confounding effects of long-lasting antidiabetic treatments and of the limited impact of duration and severity of hyperglycemia *per se* on the glucose insulin homeostatic system

## Methods

### Verona Newly Diagnosed Type 2 Diabetes Study (VNDS)

The VNDS is an ongoing study aiming at building a biobank of patients with newly diagnosed type 2 diabetes [9], [10]. A detailed description of the study population and of the experimental design is found online. In this study we report the data collected in 595 patients, whose characteristics are summarized in Table 1. Each subject gave informed written consent before participating in the research, which was approved by the Human Investigation Committee of the Verona City Hospital.

**Table 1:** Clinical and metabolic features of the VNDS population.

Variable	Males	Females	All
Number (M/F)	409	186	595
Age (yrs)	59 [51–65]	61 [56–67]	60 [52–66]
BMI (kg/m2)	28.7 [26.1–32.1]	30.4 [27.2–34.1]	29.3 [26.4–32.9]
Waist (cm)	102 [94–111]	97 [90–103]	100 [93–108]
Fasting P-glucose (mmol/l)	7 [6.2–8.0]	7.2 [6.2–8.1]	7.1 [6.2–8.0]
2hr P-glucose (mmol/l)	13.1 [10.8–16.2]	12.9 [10.1–16.3]	13.1 [10.6–16.2]
HbA1c (%)	6.7 [6.1–7.5]	6.6 [6.2–7.4]	6.6 [6.1–7.5]
Triglycerides (mmol/l)	1.5 [1.0–2.1]	1.3 [1.0–2.0]	1.4 [1.0–2.0]
HDL-cholesterol (mmol/l)	1.1 [0.95–1.3]	1.2 [1.0–1.4]	1.1 [1.0–1.3]
Cholesterol (mmol/l)	4.8 [4.2–5.5]	5.1 [4.5–5.8]	5.0 [4.3–5.6]
SBP (mmHg)	134 [120–146]	140 [130–150]	136 [122–150]
DBP (mmHg)	80 [80–90]	85 [80–90]	82 [80–90]
Insulin Sensitivity (µmol/min/m2 BSA)	602 [365–885]	596 [408–796]	599 [384–856]
Insulinogenic Index (mU/mmol)	3.6 [2.0–5.9]	4.8 [2.5–8.2]	3.9 [2.1–6.9]
CIR120′ (mUxL/mmol2)	0.4 [0.2–1.1]	0.6 [0.3–1.4]	0.5 [0.2–1.3]

Data are presented as median [I.Q. range].

Standard clinical parameters were assessed in all patients. Metabolic tests were carried out on two separate days in random order. On one day an OGTT (75 g) was performed to assess beta cell function, as previously described [11]. On a separate day, a euglycemic insulin clamp was performed to assess insulin sensitivity [9]

### Analytical procedures

Plasma glucose concentration was measured in duplicate with a Beckman Glucose Analyzer II (Beckman Instruments, Fullerton, CA, USA) or a YSI 2300 Stat Plus Glucose&Lactate Analyzer (YSI Inc., Yellow Springs, OH, USA), at bedside. Serum C-peptide and insulin concentrations were measured by chemiluminescence as previously described [12]. Glycated hemoglobin and serum lipids were measured by standard in-house methods. GAD-antibodies were measured by immunoradiometry (CentAK, Medipan, Germany), according to manufacturer's instructions.

### Calculations

The amount of glucose metabolized during the last 60 min of the clamp (M value, reference insulin sensitivity) was computed with standard formulae [13], [14].

The following classical indexes of beta cell function were computed [15]:

- Insulinogenic Index: (Insulin_30′_−Insulin_0′_)/(Glucose_30′_−Glucose_0′_), units: mU/mmol;
- Corrected Insulin Response_120′_ (CIR_120′_): Insulin_120′_/[Glucose_120′_*(Glucose_120′_−3.89)] [15]; units: mU*L/mmol^2^.

Further insights in beta cell function were sought by mathematical modeling (see below and Supporting Information S1 Tables S1, S2).

### Modeling of Beta Cell Function

The analysis of the glucose and C-peptide curves during the OGTTs of the VNDS was performed as described in previous publications [9], [10] and builds upon previous works from other laboratories [16], [17]. Insulin secretion rate is described as the sum of two components, one responding to the rate of increase of glucose, the other to glucose concentration itself. Modeling details are found online in the Supporting Information S1.

There are two main physiological outputs of the model:

1. derivative control [pmol·m^−2^ BSA] · [mmol·l^−1^·min^−1^] ^−1^ of beta cell function: it is presented as the amount of insulin secreted in response to a rate of glucose increase of 1 mmol/l per min which lasts for 1 minute;
2. proportional control of beta cell function: it is presented as the stimulus-response curve linking glucose concentration (x axis) to insulin secretion rate (ISR [pmol·min^−1^·m^−2^ BSA]; y axis) at the pre-selected glucose concentrations of 5.5, 8.0, 11.0, 15.0 and 20.0 mmol/l.

### Genotyping

A peripheral blood sample was collected from each patient and the DNA was extracted by standard salting out method.

**Figure 1:**
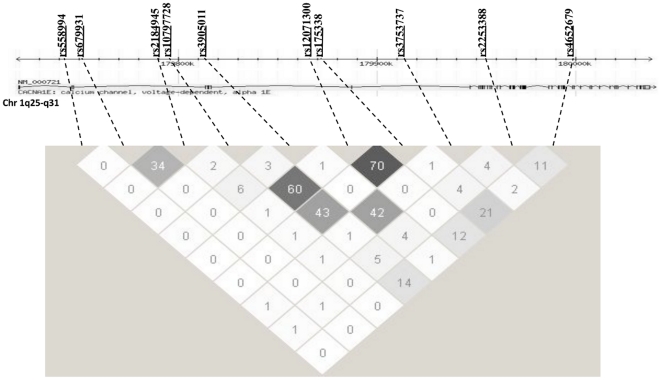
Gene structure, location of polymorphic sites, and pairwise LD among SNPs in *CACNA1E*. The upper portion of the figure shows the gene structure and location of the polymorphisms genotyped in the VNDS population. The lower portion of the figure shows a schematic of the pairwise LD, calculated as *r* ^2^, among the SNPs in the VNDS patients. The dotted lines connect each SNP name and position with the corresponding cell in the LD matrix. Increasing level of LD is shown by darker grayscale.

Ten tag-SNPs (rs558994, rs679931, rs2184945, rs10797728, rs3905011, rs12071300, rs175338, rs3753737, rs2253388 and rs4652679), which capture ∼93% of the common genetic variability of this genomic region (about 320 kbp) (fig. 1), were selected with the software GEVALT (GEnotype Visualization and ALgorithmic Tool) [18]. The LD values in our population, expressed as r^2^, are consistent with the values reported by HapMap (fig. 1).

Genotypes were assessed by the high-throughput genotyping Veracode technique (Illumina Inc, CA), applying the GoldenGate Genotyping Assay according to manufacturer's instructions [19].

### Statistical Analysis

Data are presented as medians and interquartile range, unless otherwise indicated. Hardy-Weinberg equilibrium was tested by chi-square test. Before comparisons, skewed variables were log-transformed to improve the approximation to the Gaussian distribution. Single variant association analyses were carried out by generalized linear models (GLM) as implemented in SPSS and they were adjusted for a number of potential confounders, including age, gender and BMI. Since the logarithmic transformation was insufficient to improve the distribution of the derivative control of beta cell function, the latter was first analyzed by Kruskal-Wallis test. All statistical analyses were carried out with the SPSS 12.0 software. Statistical significance was declared at p<0.05.

## Results

### Clinical Features of the Study Population

Anthropometric, clinical and metabolic features of the study subjects are shown in table 1. Further details can be found in the Supporting Information S1.

### Effects of CACNA1E variants on clinical and metabolic parameters

Allele distribution was compatible with the Hardy-Weinberg equilibrium in all 10 SNPs (p>0.16 or more).

Anthropometric, clinical and metabolic features of the study subjects, layered according to rs558994, rs679931, rs2184945, rs10797728, rs3905011, rs12071300, rs175338, rs3753737, rs2253388 and rs4652679 alleles, are found in the online material (Supporting Information S1 Tables from S3 to S12).

### Effects of CACNA1E variants on derivative and proportional control of beta cell function

Both major alleles of rs10797728 (*A*) and rs175338 (*G*), which are in modest linkage disequilibrium with each other (D′: 0.9, r^2^: 0.43) (fig. 1), were associated to a significant impairment in the derivative control of beta cell function, i.e. the response of beta cell to the rate of glucose increase (p = 0.01 and p = 0.03 respectively by Kruskal-Wallis) (Table 2). The major allele of rs10797728 was also associated to lower basal insulin secretion rate (−0.07±0.03 log units, p<0.03) (Supporting Information S1 Table S6).

**Table 2:** Derivative control of beta cell function in patients of the VNDS according to genotype of *CACNA1E* variants.

*CACNA1E* SNP	n	AA	AB	BB	*p* value(k-w)
rs558994	514	653.6±47.6	609.9±40.4	568.2±105.3	0.69
rs679931	515	700.1±50.9	578.2±42.3	712±125.4	0.11
rs2184945	521	707.1±54.6	585.5±46.7	683.1±71.2	**0.04**
rs10797728	523	658±42.1	558±47.5	1015±209.8	**0.01**
rs3905011	521	580.5±46.3	646.9±46.2	734.7±96.4	0.59
rs12071300	529	642.9±38	587±57.4	962.5±205.1	0.07
rs175338	507	658.8±41.5	570.3±49.1	1054.3±218.7	**0.03**
rs3753737	512	636.1±42.4	656.6±55	620.8±94.2	0.91
rs2253388	515	619.5±43.7	641.2±51.4	754±111.1	0.32
rs4652679	518	629.2±42.7	636.5±47.9	598.6±118.7	0.85

Data are presented as mean±SE. A non-parametric test has been performed (Kruskal-Wallis) since the variable was not normally distributed.

A = major allele; B = minor allele.

Patients carrying the major alleles *A* of rs2184945, under a dominant model, (Supporting Information S2 Fig. S1a) or *G* of rs3905011 (Supporting Information S2 Fig. S1b) had defective proportional control of beta cell function, i.e. the curve relating glucose (stimulus) to insulin secretion rate (response) (p<0.01 and p<0.005 respectively by ANOVA). The effects of rs2184945 and rs3905011 genotypes on the beta cell response to glucose concentration were statistically independent of each other (p<0.02 and p<0.01, respectively, in a multivariate model including both SNPs), consistently with their low degree of linkage disequilibrium (D′: 0.26, r^2^: 0.06).

**Figure 2:**
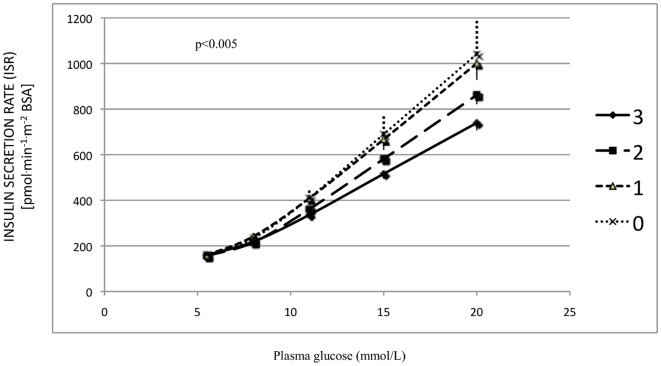
Effects of *CACNA1E* score on the curve relating insulin secretion rate (y axis) to glucose concentration (x axis), i.e. the proportional control of beta cell function, in patients with newly diagnosed type 2 diabetes of the VNDS. The *CACNA1E* score was computed by considering two levels for rs2184945 (*TT* and *AA*/*AT*) and 3 levels for rs3905011 (*AA*, *GA* and *GG*) and by scoring them from 0 to 1 or 2 respectively. The *CACNA1E* score could range from a minimum of 0 (double genotype rs2184945*TT*–*rs3905011AA*) to a maximum of 3 (a *rs2184945AA*/*AT*–rs3905011*GG* genotype) (Supporting Information S1 Table S13). The higher was the *CACNA1E* score, the lower was the beta cell insulin secretory response to glucose (p<0.005 after adjusting for age, gender and BMI). Data are presented as mean±SEM.

We then computed a *CACNA1E* score in order to investigate the combined effect of rs2184945 and rs3905011 on the proportional control, by assigning a score of 1 to all carriers of the rs2184945 *A* allele, irrespectively of zygosity, and a score of 1 for each rs3905011 *G* allele. Hence, the *CACNA1E* score could range from a minimum of 0 (rs2184945 *TT*–rs3905011 *AA* genotype) to a maximum of 3 (genotype rs2184945 *AA* or *AT*–rs3905011 *GG* genotype) (Supporting Information S1 Table S13). The higher was the *CACNA1E* score, the lower was the insulin secretion rate in response to glucose concentration (Fig. 2) (p<0.005).

Finally, the major *G* allele of rs4652679 was associated to significantly lower basal insulin secretion rate (−0.06±0.03 log units, p<0.04) and to significantly more efficient insulin clearance (+0.07±0.02 log units, p<0.01) (Supporting Information S1 Table S12).

### Effects of CACNA1E variants on surrogate indexes of beta cell function

The above results were mirrored by significantly lower CIR_120_, a surrogate index of beta cell function, both in carriers of either rs2184945 *A* allele or rs3905011 *G* allele and in carriers of progressively higher *CACNA1E* score (+0.23±0.06 log units, p<0.001, data not shown).

Furthermore, lower fasting and 2-hour insulin levels were associated to the rs3905011 *G* allele (Supporting Information S1 Table S7). In the latter case, statistical significance stayed the same, even after adjusting for basal insulin secretion and clamp-measured insulin clearance (Supporting Information S1 Table S7).

### Effect of CACNA1E variants on insulin sensitivity and indicators of glucose control

The minor *A* allele of rs2253388 was associated to more severe insulin resistance, according to a recessive model (+0.08±0.04 log units p<0.05) (Supporting Information S1 Table S11). This association stayed statistically significant after adjusting for age, gender and BMI.

In parallel to their influence on beta cell function, the rs2184945 *A* allele and the rs3905011 *G* allele were associated to higher 2-hour plasma glucose (both SNPs) and to worse fasting plasma glucose and HbA1c (rs3905011 *G*) (Supporting Information S1 Tables S5 and S7).

Further associations of CACNA1E variability with other clinical metabolic parameters are reported and discussed in the Supporting Information S1.

## Discussion

This study explored the potential role(s) of ten *CACNA1E* variants (fig. 1), purposely selected to capture ∼93% of common *CACNA1E* variability, in determining pathophysiological phenotypes (i.e. beta cell function and insulin sensitivity) in patients with newly diagnosed type 2 diabetes of the VNDS. We also genotyped rs679931 and rs3753737 because of their previously reported role in type 2 diabetes risk and in beta cell function regulation [7], [8].

The interesting and novel feature of our study is the use of state-of-art methods [16], [17], [20], [21], instead of surrogate markers, to assess beta cell function and insulin sensitivity in a sample of patients with newly diagnosed type 2 diabetes. This population is presumably not yet influenced by long-standing hyperglycemia and/or pharmacological antidiabetic treatment, which could modify phenotype-genotype interaction.

Our data show that up to five (rs10797728, rs175338, rs2184945, rs3905011 and rs4652679) out of ten *CACNA1E* SNPs may exert some influence on different components of beta cell function (derivative control and proportional control), which in some (rs2184945 and rs3905011 genotypes), but not all cases, are also reflected by changes in glucose control (Supporting Information S1 Tables S5 and S7).

The apparent influence exerted by rs10797728 and 175338 on the derivative control of beta cell function (table 2) is somewhat surprising, in the light of the claim that Ca_V_2.3 is involved in the regulation of only second phase insulin secretion [3] and that the derivative control of beta cell function describes first phase insulin secretion in response to intravenous glucose challenges [11], [20], [21]. This apparent lack of biological plausibility may indicate that this is simply a chance finding. However, at a close inspection, the data reported by Jing et al., possibly the most thorough study on the relationship between Ca_V_2.3 activity and beta cell function, do show a trend also for reduced first phase insulin secretion in perfused pancreata from Ca_V_2.3^−/−^ mice [3]. Thus, if replicated in future studies, this finding may be of pathophysiological relevance in patients with newly diagnosed type 2 diabetes.

Both rs2184945 and rs3905011 genotypes exerted a strong, independent influence on the proportional control of beta cell function (Supporting Information S2 fig. S1a and fig. S1b, respectively). Furthermore, our finding that homozygous carriers of “poor” beta cell function alleles at both SNPs had a ∼30% reduction in the insulin secretory response to glucose, compares well with the 20–30% reduction of second phase insulin secretion reported in perfused pancreata from Ca_V_2.3^−/−^ mice [3]. It is noteworthy that the p value of the *CACNA1E* score was virtually unaffected (p = 0.001) by the inclusion in a multivariate model also of the genetic variants previously reported by us to be associated to beta cell function in the VNDS (rs7901695 or rs7903146 in *TCF7L2* and rs6717980 or rs2384628 in *GCKR*) [9], [10]. Furthermore, the associations of these two SNPs with indicators of glucose control, such as plasma glucose and HbA1c, are consistent with a potentially relevant role in glucose homeostasis of patients with newly diagnosed type 2 diabetes (Supporting Information S1 Tables S5 and S7).

In the present study, we did not genotype the +8130A/G variant (rs41315711) of *CACNA1E*, previously reported to be associated to diabetes risk and insulin resistance in Pima Indians [7], or any variant in linkage disequilibrium with it. This prevented us from replicating in the VNDS its association with insulin sensitivity. Nevertheless, the admittedly weak association of the rs225338 A allele with worse insulin resistance we report in our study (Supporting Information S1 Table S11) may reinforce the rationale to investigate more thoroughly the relationship of this gene, or of a closely related region, with the mechanisms modulating insulin sensitivity. Since, according to the present body of knowledge, Ca_V_2.3 plays direct pathophysiological roles in the beta cell [3], [6], the relationship of its genetic variability to insulin resistance, if not spurious, has to be an indirect one.

Our findings may have two further implications. First, as already shown by us for *TCF7L2* [9] and *GCKR* [10], a role of *CACNA1E* in determining beta cell function can be detected also in overt diabetes. Thus, recognizing and assessing genetic heterogeneity in patients with type 2 diabetes might turn out to be valuable in outlining a “metabolic” prognosis. Second, different genomic regions of the candidate gene *CACNA1E* are involved in regulating different components of beta cell function, suggesting that this genomic area, or genomic areas closely related to it, may play a relevant role in regulating both basal and glucose induced insulin secretion.

There are a number of limitations in our study. i- This is not a population based study, although we found that VNDS subjects are fairly representative of Italian patients with type 2 diabetes, comparing of their features with previously published studies [22]. However, in our cohort there is a higher than expected prevalence of the male gender, possibly reflecting a gender related referral bias. ii. The size of our cohort is not very large, although the direct assessment of the phenotypes of interest, instead of using surrogate indexes, may partially compensate for it. iii- We did not explore many important pathophysiological phenotypes, e.g. incretin role and liver vs peripheral insulin resistance, which are not measured by the state-of-art methods that we used to assess beta cell function and insulin sensitivity [16], [17], [20], [21]. iv- Our findings need to be replicated in an independent sample of patients, as in all association studies. This may prove difficult, because generating direct measures of beta cell function and insulin sensitivity in large samples requires time and considerable resources. Indeed, to the best of our knowledge, there is no other large cohort of European patients with type 2 diabetes who underwent the same kind of metabolic phenotyping. v- Our study was performed in Italian patients; extrapolations to other ethnic groups should be made with caution.

In summary, we have reported that common *CACNA1E* variation is significantly related to beta cell function in patients with newly diagnosed type 2 diabetes. Hence, we speculate that *CACNA1E* genotype might be a useful tool to refine diagnosis and prognosis of patients with type 2 diabetes.

## Supporting Information

Supporting Information S1 Includes text with supplemental information regarding methods and results, and tables from S1 to S13.
(DOC)

Supporting Information S2 Includes supplemental figures S1a and S1b.
(DOC)

## References

[1] Yang SN, Berggren PO (2005). CaV2.3 channel and PKClambda: new players in insulin secretion.. J Clin Invest.

[2] Williams ME, Marubio LM, Deal CR, Hans M, Brust PF (1994). Structure and functional characterization of neuronal alpha 1E calcium channel subtypes.. J Biol Chem.

[3] Jing X, Li DQ, Olofsson CS, Salehi A, Surve VV (2005). CaV2.3 calcium channels control second-phase insulin release.. J Clin Invest.

[4] Pereverzev A, Vajna R, Pfitzer G, Hescheler J, Klockner U (2002). Reduction of insulin secretion in the insulinoma cell line INS-1 by overexpression of a Ca(v)2.3 (alpha1E) calcium channel antisense cassette.. Eur J Endocrinol.

[5] Matsuda Y, Saegusa H, Zong S, Noda T, Tanabe T (2001). Mice lacking Ca(v)2.3 (alpha1E) calcium channel exhibit hyperglycemia.. Biochem Biophys Res Commun.

[6] Pereverzev A, Mikhna M, Vajna R, Gissel C, Henry M (2002). Disturbances in glucose-tolerance, insulin-release, and stress-induced hyperglycemia upon disruption of the Ca(v)2.3 (alpha 1E) subunit of voltage-gated Ca(2+) channels.. Mol Endocrinol.

[7] Muller YL, Hanson RL, Zimmerman C, Harper I, Sutherland J (2007). Variants in the Ca V 2.3 (alpha 1E) subunit of voltage-activated Ca2+ channels are associated with insulin resistance and type 2 diabetes in Pima Indians.. Diabetes.

[8] Holmkvist J, Tojjar D, Almgren P, Lyssenko V, Lindgren CM (2007). Polymorphisms in the gene encoding the voltage-dependent Ca(2+) channel Ca (V)2.3 (CACNA1E) are associated with type 2 diabetes and impaired insulin secretion.. Diabetologia.

[9] Bonetti S, Trombetta M, Malerba G, Boselli L, Trabetti E (2011). Variants and haplotypes of TCF7L2 are associated with beta-cell function in patients with newly diagnosed type 2 diabetes: the Verona Newly Diagnosed Type 2 Diabetes Study (VNDS) 1.. J Clin Endocrinol Metab.

[10] Bonetti S, Trombetta M, Boselli ML, Turrini F, Malerba G (2011). Variants of GCKR Affect Both {beta}-Cell and Kidney Function in Patients With Newly Diagnosed Type 2 Diabetes: The Verona Newly Diagnosed Type 2 Diabetes Study 2.. Diabetes Care.

[11] Bonadonna RC, Heise T, Arbet-Engels C, Kapitza C, Avogaro A (2010). Piragliatin (RO4389620), a novel glucokinase activator, lowers plasma glucose both in the postabsorptive state and after a glucose challenge in patients with type 2 diabetes mellitus: a mechanistic study.. J Clin Endocrinol Metab.

[12] Cretti A, Lehtovirta M, Bonora E, Brunato B, Zenti MG (2001). Assessment of beta-cell function during the oral glucose tolerance test by a minimal model of insulin secretion.. Eur J Clin Invest.

[13] Targher G, Bonadonna RC, Alberiche M, Zenere MB, Muggeo M (2001). Relation between soluble adhesion molecules and insulin sensitivity in type 2 diabetic individuals: role of adipose tissue.. Diabetes Care.

[14] Saccomani MP, Bonadonna RC, Bier DM, DeFronzo RA, Cobelli C (1996). A model to measure insulin effects on glucose transport and phosphorylation in muscle: a three-tracer study.. Am J Physiol.

[15] Hanson RL, Pratley RE, Bogardus C, Narayan KM, Roumain JM (2000). Evaluation of simple indices of insulin sensitivity and insulin secretion for use in epidemiologic studies.. Am J Epidemiol.

[16] Cobelli C, Toffolo GM, Dalla Man C, Campioni M, Denti P (2007). Assessment of beta-cell function in humans, simultaneously with insulin sensitivity and hepatic extraction, from intravenous and oral glucose tests.. Am J Physiol Endocrinol Metab.

[17] Mari A, Camastra S, Toschi E, Giancaterini A, Gastaldelli A (2001). A model for glucose control of insulin secretion during 24 h of free living.. Diabetes.

[18] Davidovich O, Kimmel G, Shamir R (2007). GEVALT: an integrated software tool for genotype analysis.. BMC Bioinformatics.

[19] Lin CH, Yeakley JM, McDaniel TK, Shen R (2009). Medium- to high-throughput SNP genotyping using VeraCode microbeads.. Methods Mol Biol.

[20] Cali AM, Bonadonna RC, Trombetta M, Weiss R, Caprio S (2008). Metabolic abnormalities underlying the different prediabetic phenotypes in obese adolescents.. J Clin Endocrinol Metab.

[21] Weiss R, Caprio S, Trombetta M, Taksali SE, Tamborlane WV (2005). Beta-cell function across the spectrum of glucose tolerance in obese youth.. Diabetes.

[22] Bonora E, Targher G, Formentini G, Calcaterra F, Lombardi S (2004). The Metabolic Syndrome is an independent predictor of cardiovascular disease in Type 2 diabetic subjects. Prospective data from the Verona Diabetes Complications Study.. Diabet Med.

