# Supplementary material for: CACNA1E Variants Affect Beta Cell Function in Patients with Newly Diagnosed Type 2 Diabetes. The Verona Newly Diagnosed Type 2 Diabetes Study (VNDS) 3
Source: PLoS One. 2012 Mar 9;7(3):e32755. doi: 10.1371/journal.pone.0032755 (PMC3302892; doi:10.1371/journal.pone.0032755)
Supplement: Supporting Information S1 — Includes text with supplemental information regarding methods and results, and tables from S1 to S13. (DOC) [file pone.0032755.s001.doc]

**Supporting Information S1**

**Variants of *CACNA1E* affect beta cell function in patients with newly diagnosed type 2 diabetes.**

**The Verona Newly Diagnosed Type 2 Diabetes Study (VNDS) 3.**

Maddalena Trombetta^1,2^**^§^**, Sara Bonetti^1^**^§^**, MariaLinda Boselli^1^, Fabiola Turrini^1^, Giovanni Malerba^3^, Elisabetta Trabetti^3^, PierFranco Pignatti^3^, Enzo Bonora^1, 2^, Riccardo C. Bonadonna^1, 2^

**Institution**: ^1^ Department of Medicine, University of Verona, Verona, Italy; ^2^ Azienda Ospedaliera Universitaria Integrata di Verona, Verona, Italy; ^3^ Department of Life and Reproduction Sciences, University of Verona, Verona, Italy

**^§^** These Authors contributed equally to this paper

**Experimental Design**

The Verona Newly Diagnosed Type 2 Diabetes Study (VNDS) is an ongoing study aiming at building a biobank of patients with newly diagnosed type 2 diabetes. As of Jan 1 2002, all patients referred to the Division of Endocrinology and Metabolic Diseases of University of Verona School of Medicine, whose diabetes has been diagnosed in the last six months, are asked to participate in this research. The clinical evidence on which the diagnosis of type 2 diabetes has been made is reviewed and the diagnosis confirmed . Patients are drug-naïve or, if already treated with antidiabetic drugs, undergo a treatment washout of at least one week before metabolic tests are performed. Among the exclusion criteria are age>75 years, non-Italian ancestry, insulin treatment, presence of anti-GAD antibodies, malignancies, and any condition severely impairing liver and/or kidney function. In this study, we report the data collected in 595 patients, whose characteristics are summarized in Table 1.

All subjects consumed a weight-maintaining diet containing 200-250 g of carbohydrate/day for at least three days before studies. Body weight was stable in all subjects for at least 1 month before studies. No subject participated in any heavy exercise. Each subject gave informed written consent before participating in the research, which was approved by the Human Investigation Committee of the Verona City Hospital. Measurements of standard clinical phenotypes were collected in all patients. Metabolic tests were carried out on two separate days in random order. On both days, patients were admitted to the Metabolic Clinic Research Center at 07:30 after an overnight fast. All studies were carried out in a quiet, temperature controlled (22° C) room.

On one day an OGTT (75 g) was performed to assess beta cell function. For ethical reasons, the OGTT was not performed in patients presenting with FPG greater than 15 mmol/l. During the entire test patients were sitting in a comfortable cardiac chair. One teflon (21 g) venous catheter was inserted into an antecubital vein for blood sampling and kept patent with heparinized normal saline solution. After a 30’ rest to establish baseline and after collecting a 20 cc blood sample for leukocyte DNA extraction, at time = 0’ subjects ingested 75 g of glucose in 300 ml of water over 5 min. Blood samples to measure glucose, C-peptide and insulin concentrations were collected at times -10’, 0’, +15’, +30’, +45’, +60’, +90’, +120’, +150’, +180’, +210’ and +240’. In some patients further blood samples were collected at +270’ and +300’. Urines were collected to measure glycosuria.

On a separate day, a euglycemic insulin clamp was performed to assess insulin sensitivity . During the entire test patients were lying in bed. One teflon catheter was introduced into an antecubital vein for the infusion of test substances. Another teflon catheter was placed retrogradely into a wrist vein for sampling arterialized venous blood, according to the “hot box” technique. After a 30’ rest in bed to establish baseline, indirect calorimetry (at least 40’) was performed as previously described, for a companion study . At the end of calorimetric measures, baseline blood samples were collected and a standard euglycemic insulin (intravenous prime: 4.8 nmol^.^min^-1.^m^-2^ BSA; continuous infusion: 240 pmol^.^min^-1.^m^-2^ BSA) clamp was performed . Plasma glucose was allowed to decline until it reached 5.5 mmol/l, after which glucose clamping started with a glucose concentration goal of 5 mmol/l. The duration of the glucose clamp was at least of 120’, but it was prolonged, if and as needed, to ensure at least 60’ of insulin clamp at euglycemia in each patient. Timed blood samples were collected to measure hormone and substrate levels. In the last 45’ of the clamp indirect calorimetry was repeated to assess substrate oxidation and energy production rates for a companion study. Urines were collected to measure urea excretion rate.

In both metabolic tests, all blood samples were collected in pre-chilled tubes and readily spun at 1,500 g. Plasma and serum specimens were stored at –80° C.

**Mathematical Modeling of Beta Cell Function**

The analysis of the glucose and C-peptide curves during the OGTT follows the general strategy described in previous publications with some modifications and builds upon previous works from other laboratories . The kinetics of C-peptide is described with a two-compartment model, in which the two pools (1 and 2) exchange with each other and the irreversible loss of the hormone is from pool 1, the same where C-peptide concentration is measured. C-peptide kinetic parameters are computed according to the equations by Van Cauter et al. .

Herein are the equations describing the model of glucose induced insulin secretion during an OGTT:

*d*cp_1_(t)/*d*t = ISR(t) + cp_2_. k_12_ – (k_01_ + k_21_) . cp_1_   (*Eq.1*)

where ISR = insulin secretion rate, cp_1_ = C-peptide mass in the sampling (accessible) compartment, cp_2_ = C-peptide mass in the remote compartment, k_12_ and k_21_ = rate constants of the exchange between the two C-peptide compartments, and k_01_ = rate constant of the irreversible loss of C-peptide from the accessible compartment. Note that the values of the volume of distribution of C-peptide pool 1 (accessible compartment), k_12_, k_21_, and k_01_ are computed according to the equations by Van Cauter et al. .

ISR(t) = BSR + DSR (t) + PSR (t) (*Eq.2*)

where BSR = basal insulin secretion rate, DSR = insulin secretion rate due to the derivative (or dynamic) component, and PSR = insulin secretion rate due the proportional (or static) component.

BSR = CP_ss_ . V_1_ . k_01_ (*Eq. 3*)

where CP_ss_ is basal C-peptide concentration and V_1_ is the volume of the accessible compartment of C-peptide.

From the modeling viewpoint, DSR(t) and PSR(t) are the components which in intravenous glucose tolerance tests or hyperglycemic clamps describe classical first phase insulin secretion and second phase insulin secretion, respectively. Furthermore, from a physiological viewpoint, the sum of BSR and PSR(t) describes the relationship linking glucose concentration and insulin secretion rate, in the absence of the derivative component (DSR).

DSR(t) and PSR(t) are mathematically defined as follows:

DSR (t) = X1 (t) . t^-1^  (*Eq. 4*)

*d*X1 (t) / *d*t = s1 . [*d*G(t)/*d*t]/[log(1.1+ t)] - X1(t) . t^-1^  if *d*G(t)/*d*t > 0 (*Eq. 5*)

*d*X1(t) / *d*t = - X1(t) . t^-1^        if *d*G(t)/*d*t ≤ 0 (*Eq. 6*)

where s1 = glucose sensitivity of derivative control of insulin secretion, G = plasma glucose concentration, X1 = C-peptide (insulin) mass made available for the derivative component of insulin secretion, t = time constant of the derivative component of insulin secretion, and the term log(1.1 + t) accomodates the time-associated decline of s1 documented in humans during a hyperglycemic stimulus .

PSR(t) = X2(t) . d^-1^  (*Eq. 7*)

*d*X2(t) / *d*t = s2 . [G(t) – q] - X2 (t) . d^-1^  (*Eq. 8*)

where  s2 = glucose sensitivity of the proportional component of insulin secretion, X2 = C-peptide (insulin) mass made available for the proportional component of insulin secretion, d = time constant of the proportional component of insulin secretion, q = glucose threshold above which the beta-cell responds with the proportional component of insulin secretion to plasma glucose concentration.

This model was implemented in the SAAM 1.2 software (SAAM Institute, Seattle, WA) to estimate its unknown parameters. Numerical values of the unknown parameters were estimated by using nonlinear least squares. Weights were chosen optimally, i.e., equal to the inverse of the variance of the measurement errors, which were assumed to be additive, uncorrelated, with zero mean, and a coefficient of variation (CV) of 6-8%. The unknown parameters of the model are: CP_ss_, s1, t, s2, d, and q. They were estimated with good precision, as shown by their CVs (table S1)

A good fit of the model to data was obtained as shown by the table of the weighted residuals (table S2).

There are two main physiological outputs of the model:

1. derivative control (units: [pmol.m^-2^ BSA] . [mmol.l^-1^.min^-1^] ^–1^): it is the amount of insulin secreted in response to a rate of glucose increase of 1 mmol/l per min which lasts for 1 minute;
2. stimulus-response curve linking glucose concentration (x axis) to insulin secretion rate (y axis): as explained above, it is the sum of BSR and PSR. With the purpose of avoiding artifactual increases in the power of statistical analyses, we used the stimulus-response curve at the pre-determined glucose concentrations of 5.5, 8.0, 11.0, 15.0 and 20.0 mmol/l.

**RESULTS**

*Clinical features of the study population*

Most patients were overweight or obese, but less than 50% were obese. However, abdominal obesity was present in ˜50% of males and in >75% of female patients. Glucose control, as measured by HbA1c, was fairly good with 25% patients having HbA1c > 7.4%. High triglyceride and low HDL-cholesterol levels were common, especially the latter one in the female patients. Systolic and diastolic hypertensive values were detected in 50% and 25% of the patients, respectively.

*Other associations of CACNA1E variants to metabolic phenotypes*

Carriers of the diabetes risk alleles *T* of rs679931 and *A* of rs3753737 had higher cholesterol levels (tables S4 and 10; p <0.02 and p<0.04, respectively). Also the major allele *G* of rs4652679 was associated to higher cholesterol levels (p<0.02) (table S12). The major alleles *G* of rs175338 and *G* of rs2253388 were associated to lower HDL-cholesterol levels (tables S9 and 11; p<0.01 and p<0.02, respectively). The major *A* allele of rs2184549 was associated to decreased diastolic blood pressure (table S5; p<0.04).

**DISCUSSION**

A cluster of nominally statistically significant associations between cholesterol levels and *CACNA1E* genetic variability has been found in our cohort of patients with newly diagnosed type 2 diabetes. Although the number of these associations seems to rule out the possibility of statistical significances due to chance alone, these results are to be considered with extreme caution, the more so because of the lack of sufficient biological plausibility.

The association between one *CACNA1E* SNP and diastolic blood pressure may be in line with a chance finding.

**Table S1**. Coefficients of variation of the beta cell model parameters. CP_ss_= basal C-peptide concentration; σ1 = parameter regulating glucose sensitivity of derivative control of insulin secretion, τ = time constant of derivative control of insulin secretion, σ2 = glucose sensitivity of proportional control of insulin secretion, δ = time constant of proportional control of insulin secretion, θ: glycemic threshold of proportional control of insulin secretion.

|  | **Coefficients of Variation (%)** | |
| --- | --- | --- |
| **Model Parameter** | **Median** | **I.Q. Range** |
| CP_ss_ | 10.8 | 6.9-18.3 |
| σ1 | 40.7 | 24.2-83.4 |
| τ | 60.3 | 58.2-61.2 |
| σ2 | 16.2 | 11.9-22.4 |
| δ | 33.0 | 21.4-66.7 |
| θ | 13.4 | 8.5-22.0 |

**Table S2**. Weighted residuals of the model fit to the C-peptide data of the OGTT. Data are means±SD. The weighted residuals are a quantitative point-by-point assessment of the goodness-of-fit of the model to the data: a theorically perfect fit should generate weighted residuals with mean 0 and SD of 1.

|  | C-Peptide weighted residuals | | | | | | | | | |
| --- | --- | --- | --- | --- | --- | --- | --- | --- | --- | --- |
| Time | 15’ | 30’ | 45’ | 60’ | 90’ | 120’ | 150’ | 180’ | 210’ | 240’ |
| **Mean** | -0.397 | +0.118 | +0.199 | +0.267 | +0.115 | -0.026 | +0.142 | +0.015 | +0.026 | +0.107 |
| **SD** | 1.03 | 1.14 | 1.197 | 1.263 | 1.302 | 1.213 | 1.319 | 1.268 | 1.277 | 1.164 |

Table S3. Influence of rs558994 genotype on clinical and metabolic variables of patients with newly diagnosed type 2 diabetes in the VNDS. Beta and SE are the multivariate model estimates of the change and its standard deviation, respectively, in the phenotypic trait associated to the presence of one minor allele. Reported p values were obtained after adjusting for age, gender and BMI. *Variables were log-transformed before statistical analysis to approximate a gaussian distribution; the coefficient beta and its S.E. refer to log-transformed values.

|  | | **rs558994** | | | **Additive model** | | |
| --- | --- | --- | --- | --- | --- | --- | --- |
| **Phenotype** | **N** | **GG** | **AG** | **AA** | **BETA** | **SE** | ***P* Value** |
| **Minor allele frequency, %** | | **30** | | |  |  |  |
| Number (M/F) | 580  (399/181) | 275 | 258 | 47 |  |  |  |
| Age (yrs) | 580 | 59 [52-65] | 60 [52-66] | 60 [53-64] | -0.003 | 0.67 | 0.99 |
| BMI* (kg/m^2^) | 580 | 29.4 [26.4-33.1] | 29.2 [26.4-32.2] | 2.7 [26.7-34.7] | -0.003* | 0.01* | 0.78 |
| Fasting P-glucose (mmol/l) | 545 | 7 [6.0-7.9] | 7.2 [6.4-8.1] | 6.8 [6.1-8.1] | -0.08 | 0.12 | 0.51 |
| 2hr P-glucose (mmol/l) | 534 | 12.9 [10.3-15.9] | 13.7 [10.9-16.6] | 12.8 [10.3-15.7] | 0.04 | 0.28 | 0.88 |
| Fasting insulin* (mU/l) | 468 | 11.2 [7.3-16.3] | 11.6 [7.6-16.3] | 11.1 [7.8-15.2] | 0.04* | 0.04* | 0.34 |
| 2hr insulin* (mU/l) | 480 | 62.5[36.5-100] | 61.2 [39.2-95.8] | 73.2 [44.5-113] | 0.05* | 0.05* | 0.30 |
| Insulinogenic Index (mU/mmol)* | 431 | 3.5 [2-8.2] | 4.2 [2.2-5.9] | 4 [2.5-6.2] | -0.04* | 0.07* | 0.55 |
| CIR_120’_ *(mUxL/mmol^2^) | 479 | 0.5 [0.2-1.4] | 0.5 [0.2-1.1] | 0.6 [0.3-1.3] | 0.02* | 0.09* | 0.84 |
| HbA1c (%) | 556 | 6.6 [6.1-7.4] | 6.7 [6.2-7.6] | 6.5 [6-7] | -0.006 | 0.09 | 0.94 |
| Triglycerides* (mmol/l) | 562 | 1.4 [1-2.1] | 1.4 [1-2] | 1.3 [1-1.8] | 0.01* | 0.03* | 0.67 |
| HDL-cholesterol* (mmol/l) | 555 | 1.1 [1-1.3] | 1.2 [1-1.4] | 1.1 [1-1.4] | 0.02* | 0.02* | 0.25 |
| Cholesterol (mmol/l) | 562 | 4.9 [4.2-5.6] | 5 [4.4-5.7] | 4.9 [4.4-5.3] | 0.03 | 0.07 | 0.67 |
| SBP (mmHg) | 562 | 136 [124-150] | 138 [120-150] | 131 [128-150] | -0.38 | 1.17 | 0.75 |
| DBP (mmHg) | 562 | 82 [80-90] | 82 [80-90] | 87 [80-90] | -0.06 | 0.62 | 0.93 |
| Basal secretion rate* | 475 | 136.4 [97.1-175.8] | 129.8 [100.1-171.8] | 145.3 [105.7-203.7] | 0.04* | 0.03* | 0.16 |
| Insulin clearance* ml/min/m^2^ BSA | 486 | 696 [543-831] | 687 [534-799] | 715 [591-811] | 0.004* | 0.02* | 0.85 |
| Insulin Sensitivity*  (µmol/min/m^2^ BSA) | 561 | 605 [356-899] | 572 [384-823] | 653 [449-797] | 0.03* | 0.04* | 0.48 |

Table S4. Influence of rs679931 genotype on clinical and metabolic variables of patients with newly diagnosed type 2 diabetes in the VNDS. Beta and SE are the multivariate model estimates of the change and its standard deviation, respectively, in the phenotypic trait associated to the presence of one minor allele. Reported p values were obtained after adjusting for age, gender and BMI. *Variables were log-transformed before statistical analysis to approximate a gaussian distribution; the coefficient beta and its S.E. refer to log-transformed values.

|  | | **rs679931** | | | **Additive model** | | |
| --- | --- | --- | --- | --- | --- | --- | --- |
| **Phenotype** | **N** | **AA** | **AT** | **TT** | **BETA** | **SE** | ***P* Value** |
| **Minor allele frequency, %** | | **34** | | |  |  |  |
| Number (M/F) | 581  (399/182) | 246 | 275 | 60 |  |  |  |
| Age (yrs) | 581 | 60 [52-66] | 60 [52-66] | 59 [52-65] | 0.10 | 0.65 | 0.88 |
| BMI* (kg/m^2^) | 581 | 29.1 [26-33.4] | 29.1 [26.8-32.1] | 30.8 [26.5-34] | 0.02* | 0.10* | 0.15 |
| Fasting P-glucose (mmol/l) | 545 | 7 [6.2-7.9] | 7.2 [6.2-8.3] | 6.9 [6.2-7.5] | -0.08 | 0.12 | 0.50 |
| 2hr P-glucose (mmol/l) | 534 | 12.9 [10.5-16.3] | 13.7 [10.7-16.3] | 12.2 [10.3-15] | -0.33 | 0.28 | 0.24 |
| Fasting insulin* (mU/l) | 474 | 11.1 [7.2-15.2] | 11.3 [7.7-15.9] | 13.9 [7.7-19.7] | 0.03* | 0.04* | 0.37 |
| 2hr insulin* (mU/l) | 483 | 62.6[32.8-95] | 61.7 [40-98] | 70.6 [35.3-107] | 0.04* | 0.05* | 0.45 |
| Insulinogenic Index (mU/mmol)* | 431 | 3.6 [2-6.4] | 4.1 [2.3-6.7] | 5.2 [2.2-8.7] | 0.06* | 0.07* | 0.37 |
| CIR_120’_ *(mUxL/mmol^2^) | 482 | 0.5 [0.2-1.2] | 0.5 [0.2-1.3] | 0.7 [0.4-1.3] | 0.14* | 0.08* | 0.09 |
| HbA1c | 556 | 6.7 [6.1-7.5] | 6.6 [6.2-7.4] | 6.6 [6-7.2] | -0.16 | 0.09 | 0.07 |
| Triglycerides* (mmol/l) | 563 | 1.3 [1 -2] | 1.4 [1-2] | 1.7 [1.2-2.3] | 0.06* | 0.03* | 0.06 |
| HDL-cholesterol* (mmol/l) | 556 | 1.1 [1-1.3] | 1.2 [1-1.4] | 1 [0.9-1.3] | 0.02* | 0.02* | 0.30 |
| Cholesterol (mmol/l) | 563 | 4.8 [4.1-5.6] | 5 [4.5-5.6] | 5.2 [4.2-5.8] | 0.15 | 0.06 | **0.019** |
| SBP (mmHg) | 562 | 134 [120-150] | 140 [126-150] | 136 [129-140] | 0.54 | 1.13 | 0.63 |
| DBP (mmHg) | 562 | 80 [80-90] | 84 [80-90] | 85 [80-90] | 1.06 | 0.6 | 0.08 |
| Basal insulin secretion* | 482 | 132.2 [97.2-162.9] | 137.2 [98.9-181.9] | 147.3 [108.2-180.4] | 0.04* | 0.03* | 0.19 |
| Insulin clearance* ml/min/m^2^ BSA | 526 | 690 [520-810] | 699 [564-815] | 658 [548-768] | 0.02* | 0.02* | 0.45 |
| Insulin Sensitivity*  (µmol/min/m^2^ BSA) | 562 | 604 [399-874] | 611 [367-797] | 539 [307-915] | -0.05* | 0.04* | 0.17 |

Table S5. Influence of rs2184945 genotype on clinical and metabolic variables of patients with newly diagnosed type 2 diabetes in the VNDS. Beta and SE are the multivariate model estimates of the change and its standard deviation, respectively, in the phenotypic trait associated to the presence of one minor allele. Reported p values were obtained after adjusting for age, gender and BMI. *Variables were log-transformed before statistical analysis to approximate a gaussian distribution; the coefficient beta and its S.E. refer to log-transformed values.

In brackets the values referred to the dominant (AA/AT) models.

|  | | **rs2184945** | | | **Additive model** | | |
| --- | --- | --- | --- | --- | --- | --- | --- |
| **Phenotype** | **N** | **AA** | **AT** | **TT** | **BETA** | **SE** | ***P* Value** |
| **Minor allele frequency, %** | | **43** | | |  |  |  |
| Number (M/F) | 588 (404/184) | 189 | 291 | 108 |  |  |  |
| Age (yrs) | 588 | 60 [53-65] | 60 [51-66] | 59 [53-66] | 0.02 | 0.60 | 0.98 |
| BMI* (kg/m^2^) | 588 | 29 [26.4-33] | 29.3 [26.6-32.1] | 29.7 [26-32.9] | 0.001* | 0.009* | 0.89 |
| Fasting P-glucose (mmol/l) | 552 | 7 [6.2-7.9] | 7.2 [6.2-8.3] | 6.9 [6-7.6] | -0.09 | 0.11 | 0.42 |
| 2hr P-glucose (mmol/l) | 540 | 13.2 [10.6-16.4] | 13.7 [11.1-16.4] | 11.8 [9.5-15.1] | -0.48  -1.21 | 0.26  0.45 | 0.07  **(0.008)** |
| Fasting insulin* (mU/l) | 480 | 11.1 [7.5-15.5] | 11.1 [7.5-16] | 12.5 [7.9-16.9] | 0.05* | 0.03* | 0.18 |
| 2hr insulin* (mU/l) | 487 | 64.3 [38.2-99.9] | 61.4 [37.2-95.2] | 70.6 [38.7-100] | 0.03* | 0.05* | 0.51 |
| Insulinogenic Index (mU/mmol)* | 436 | 4.0 [2.3-7.3] | 3.5 [2-5.9] | 5.2 [2.8-8.7] | 0.08* | 0.06* | 0.21 |
| CIR_120’_ *(mUxL/mmol^2^) | 486 | 0.5 [0.3-1.1] | 0.4 [0.2-1.2] | 0.7 [0.3-1.7] | 0.18*  (0.37) | 0.08*  (0.13) | **0.017**  **(0.005)** |
| HbA1c | 563 | 6.6 [6.1-7.4] | 6.7 [6.2-7.6] | 6.6 [6.1-7.2] | -0.12 | 0.08 | 0.15 |
| Triglycerides* (mmol/l) | 570 | 1.3 [1.1-1.9] | 1.4 [1-2.1] | 1.5 [1-2.1] | 0.03* | 0.03* | 0.39 |
| HDL-cholesterol*(mmol/l) | 564 | 1.1 [1-1.3] | 1.1 [1-1.4] | 1.1 [0.9-1.4] | 0.02* | 0.02* | 0.27 |
| Cholesterol (mmol/l) | 570 | 4.9 [4.2-5.6] | 5 [4.4-5.7] | 4.9 [4.2-5.5] | 0.05 | 0.06 | 0.37 |
| SBP (mmHg) | 569 | 135 [120-150] | 140 [124-150] | 135 [125-150] | 0.99 | 1.05 | 0.34 |
| DBP (mmHg) | 569 | 80 [80-90] | 82 [80-90] | 86 [80-90] | 1.14 | 0.55 | **0.039** |
| Basal insulin secretion* | 482 | 132.2 [105.3-175.1] | 134 [91.8-177.8] | 147.3 [108.2-172] | 0.02* | 0.03* | 0.55 |
| Insulin clearance* ml/min/m^2^ BSA | 534 | 694 [516-815] | 710 [573-832] | 639 [520-749] | -0.03* | 0.02* | 0.10 |
| Insulin Sensitivity*  (µmol/min/m^2^ BSA) | 569 | 604 [395-895] | 588 [360-819] | 615 [386-893] | -0.04* | 0.03* | 0.30 |

Table S6. Influence of rs10797728 genotype on clinical and metabolic variables of patients with newly diagnosed type 2 diabetes in the VNDS. Beta and SE are the multivariate model estimates of the change and its standard deviation, respectively, in the phenotypic trait associated to the presence of one minor allele. Reported p values were obtained after adjusting for age, gender and BMI. *Variables were log-transformed before statistical analysis to approximate a gaussian distribution; the coefficient beta and its S.E. refer to log-transformed values.

|  | | **rs10797728** | | | **Additive model** | | |
| --- | --- | --- | --- | --- | --- | --- | --- |
| **Phenotype** | **N** | **AA** | **AT** | **TT** | **BETA** | **SE** | ***P* Value** |
| **Minor allele frequency, %** | | **23** | | |  |  |  |
| Number (M/F) | 589  (404/185) | 347 | 218 | 24 |  |  |  |
| Age (yrs) | 589 | 60 [52-66] | 59 [52-65] | 60 [55-64] | -0.49 | 0.73 | 0.50 |
| BMI* (kg/m^2^) | 589 | 29.1 [26.2-32.8] | 29.6 [26.7-32.8] | 30.3 [27.1-34.3] | 0.02* | 0.01* | 0.052 |
| Fasting P-glucose (mmol/l) | 555 | 7 [6.1-8] | 7.2 [6.3-8.1] | 7 [6.2-7.6] | 0.06 | 0.14 | 0.68 |
| 2hr P-glucose (mmol/l) | 543 | 13 [10.4-16] | 13.7 [11.1-16.4] | 11.2 [9.1-15.9] | 0.12 | 0.32 | 0.70 |
| Fasting insulin* (mU/l) | 482 | 11.3 [7.3-17] | 11.1 [7.9-14.8] | 14.9 [10-23.3] | 0.02* | 0.04* | 0.60 |
| 2hr insulin* (mU/l) | 488 | 62.5 [37-96.8] | 60.2 [38.7-95.4] | 89.1 [70.6-175] | 0.06* | 0.06* | 0.31 |
| Insulinogenic Index (mU/mmol)* | 438 | 3.9 [2.2-6.7] | 3.7 [2-6.7] | 6.4 [3.4-11.6] | 0.03* | 0.08* | 0.70 |
| CIR_120’_ *(mUxL/mmol^2^) | 487 | 0.5 [0.2-1.3] | 0.5 [0.2-1] | 1.1 [0.5-2.3] | 0.04* | 0.09* | 0.67 |
| HbA1c | 565 | 6.6 [6.1-7.4] | 6.7 [6.2-7.6] | 6.7 [6.1-7.2] | 0.09 | 0.10 | 0.37 |
| Triglycerides* (mmol/l) | 571 | 1.4 [1-1.9] | 1.4 [1-2.1] | 1.9 [1-2.3] | 0.02* | 0.04* | 0.63 |
| HDL-cholesterol* (mmol/l) | 564 | 1.1 [1-1.3] | 1.1 [1-1.4] | 1.1 [0.9-1.4] | 0.01* | 0.02* | 0.60 |
| Cholesterol (mmol/l) | 571 | 4.9 [4.3-5.6] | 5.1 [4.3-5.7] | 5.1 [4.4-5.7] | 0.10 | 0.07 | 0.15 |
| SBP (mmHg) | 570 | 140 [121-150] | 131 [120-150] | 135 [125-150] | -1.02 | 1.28 | 0.43 |
| DBP (mmHg) | 570 | 84 [80-90] | 81 [80-90] | 85 [80-90] | -0.95 | 0.68 | 0.17 |
| Basal insulin secretion* | 483 | 131.1 [95.2-175.9] | 137.3 [107.1-174.4] | 156.6 [127.9-209.6] | 0.07* | 0.03* | **0.025** |
| Insulin clearance* ml/min/m^2^ BSA | 534 | 706 [551-828] | 685 [551-809] | 647 [518-715] | -0.04* | 0.02* | 0.082 |
| Insulin Sensitivity*  (µmol/min/m^2^ BSA) | 570 | 612 [385-870] | 588 [371-829] | 422 [269-684] | -0.04* | 0.04* | 0.38 |

Table S7. Influence of rs3905011 genotype on clinical and metabolic variables of patients with newly diagnosed type 2 diabetes in the VNDS. Beta and SE are the multivariate model estimates of the change and its standard deviation, respectively, in the phenotypic trait associated to the presence of one minor allele. Reported p values were obtained after adjusting for age, gender and BMI. *Variables were log-transformed before statistical analysis to approximate a gaussian distribution; the coefficient beta and its S.E. refer to log-transformed values.

|  | | **rs3905011** | | | **Additive model** | | |
| --- | --- | --- | --- | --- | --- | --- | --- |
| **Phenotype** | **N** | **GG** | **GA** | **AA** | **BETA** | **SE** | ***P* Value** |
| **Minor allele frequency, %** | | **40** | | |  |  |  |
| Number (M/F) | 588 (405/183) | 208 | 294 | 86 |  |  |  |
| Age (yrs) | 588 | 60 [53-66] | 59 [51-66] | 59 [51-65] | -0.73 | 0.62 | 0.24 |
| BMI* (kg/m^2^) | 588 | 29.4 [26.2-32.9] | 29.3 [26.6-33] | 28.3 [26.4-31.7] | -0.006* | 0.10* | 0.52 |
| Fasting P-glucose (mmol/l) | 552 | 7.3 [6.3-8.4] | 7 [6.2-8] | 6.8 [5.9-7.5] | -0.29 | 0.12 | **0.02** |
| 2hr P-glucose (mmol/l) | 541 | 13.6 [11.1-16.3] | 13.5 [10.7-16.2] | 12 [10-15.6] | -0.6 | 0.27 | **0.041** |
| Fasting insulin* (mU/l) | 480 | 11.6 [7.6-15.5] | 11.1 [7.6-16.4] | 12.2 [7.4-16.5] | 0.07* | 0.04* | **0.04** |
| 2hr insulin* (mU/l) | 487 | 62.1 [38.4-89.7] | 62.6 [35.3-96.7] | 74.7 [38.2-115.8] | 0.12* | 0.05* | **0.01** |
| Insulinogenic Index (mU/mmol)* | 438 | 4.1 [2.1-6.4] | 3.8 [2-6.9] | 3.7 [2.5-8.9] | 0.12* | 0.07* | 0.07 |
| CIR_120’_ *(mUxL/mmol^2^) | 486 | 0.4 [0.2-1.0] | 0.5 [0.2-1.3] | 0.7[0.3-1.6] | 0.21* | 0.08* | **0.009** |
| HbA1c | 563 | 6.7 [6.2-7.7] | 6.7 [6.1-7.4] | 6.5 [6-7] | -0.18 | 0.08 | **0.026** |
| Triglycerides* (mmol/l) | 570 | 1.4 [1.1-2] | 1.4 [1-2.1] | 1.3 [1-2.1] | -0.001* | 0.03* | 0.98 |
| HDL-cholesterol* (mmol/l) | 563 | 1.1 [1-1.3] | 1.1 [1-1.4] | 1.1 [0.9-1.4] | -0.004* | 0.02* | 0.78 |
| Cholesterol (mmol/l) | 570 | 5.1 [4.4-5.8] | 4.9 [4.3-5.5] | 4.8 [4.1-5.5] | -0.12 | 0.06 | **0.044** |
| SBP (mmHg) | 569 | 135 [120-150] | 140 [120-150] | 134 [124-150] | 1.00 | 1.09 | 0.36 |
| DBP (mmHg) | 269 | 80 [80-90] | 85 [80-90] | 80 [80-90] | 0.05 | 0.58 | 0.93 |
| Basal insulin secretion* | 481 | 136.5 [100.4-181.7] | 135.6 [100.2-172.4] | 136.9 [103-185.4] | 0.02* | 0.03* | 0.54 |
| Insulin clearance* ml/min/m^2^ BSA | 533 | 687 [517-813] | 699 [565-819] | 675 [567-795] | 0.001* | 0.02* | 0.99 |
| Insulin Sensitivity*  (µmol/min/m^2^ BSA) | 569 | 632 [437-864] | 571 [330-823] | 627 [395-898] | -0.04* | 0.04* | 0.26 |

Table S8. Influence of rs12071300 genotype on clinical and metabolic variables of patients with newly diagnosed type 2 diabetes in the VNDS. Beta and SE are the multivariate model estimates of the change and its standard deviation, respectively, in the phenotypic trait associated to the presence of one minor allele. Reported p values were obtained after adjusting for age, gender and BMI. *Variables were log-transformed before statistical analysis to approximate a gaussian distribution; the coefficient beta and its S.E. refer to log-transformed values.

|  | | **rs12071300** | | | **Additive model** | | |
| --- | --- | --- | --- | --- | --- | --- | --- |
| **Phenotype** | **N** | **AA** | **AC** | **CC** | **BETA** | **SE** | ***P* Value** |
| **Minor allele frequency, %** | | **17** | | |  |  |  |
| Number (M/F) | 595 (409/186) | 409 | 168 | 18 |  |  |  |
| Age (yrs) | 595 | 60 [52-66] | 59 [52-66] | 61 [55-65] | 0.08 | 0.78 | 0.92 |
| BMI* (kg/m^2^) | 595 | 29.2 [26.4-32.8] | 29.4 [26.6-32.9] | 29.2 [26.8-33.7] | 0.004* | 0.01* | 0.74 |
| Fasting P-glucose (mmol/l) | 560 | 7.1 [6.2-8] | 7.2 [6.2-8] | 6.7 [6-7.5] | -0.10 | 0.15 | 0.48 |
| 2hr P-glucose (mmol/l) | 549 | 13.1 [10.4-16.2] | 13.7 [11.1-16.4] | 10.6 [9.1-13.7] | -0.12 | 0.34 | 0.73 |
| Fasting insulin* (mU/l) | 487 | 11.5 [7.5-16.8] | 10.9 [8.1-14.6] | 13.5 [9.9-19.2] | 0.02* | 0.04* | 0.64 |
| 2hr insulin* (mU/l) | 493 | 63.2 [35.2-96.7] | 59.3 [40.4-95.4] | 86.4 [70.3-117] | 0.08* | 0.06* | 0.15 |
| Insulinogenic Index (mU/mmol)* | 443 | 4 [2.2-6.7] | 3.5 [1.8-6.5] | 6.9 [4.7-11.3] | 0.02* | 0.08* | 0.81 |
| CIR_120’_ *(mUxL/mmol^2^) | 492 | 0.5 [0.2-1.3] | 0.5 [0.2-0.9] | 1.1 [0.6-1.5] | 0.13* | 0.10* | 0.19 |
| HbA1c | 571 | 6.6 [6.1-7.4] | 6.8 [6.3-7.7] | 6.2 [6-6.6] | 0.02 | 0.10 | 0.83 |
| Triglycerides* (mmol/l) | 577 | 1.4 [1-2] | 1.4 [1-2.1] | 1.8 [1-2.2] | 0.01* | 0.04* | 0.88 |
| HDL-cholesterol*(mmol/l) | 570 | 1 [1.1-1.3] | 1.1 [1-1.4] | 1.2 [1-1.4] | 0.03* | 0.02* | 0.22 |
| Cholesterol (mmol/l) | 577 | 4.9 [4.3-5.6] | 4.9 [4.2-5.7] | 5.3 [4.9-5.9] | 0.10 | 0.08 | 0.21 |
| SBP (mmHg) | 576 | 138 [120-150] | 134 [120-150] | 140 [125-144] | -0.02 | 1.35 | 0.99 |
| DBP (mmHg) | 576 | 84 [80-90] | 82 [80-90] | 87 [79-90] | -0.20 | 0.72 | 0.78 |
| Basal insulin secretion* | 489 | 134 [98.2-177.1] | 137.5 [105.6-173.7] | 136 [118.8-162.7] | 0.04* | 0.03* | 0.250 |
| Insulin clearance* ml/min/m^2^ BSA | 540 | 701 [565-830] | 657 [553-797] | 706 [537-752] | -0.04* | 0.03* | 0.12 |
| Insulin Sensitivity*  (µmol/min/m^2^ BSA) | 576 | 597 [380-855] | 604 [406-889] | 645 [271-756] | -0.01* | 0.04* | 0.76 |

Table S9. Influence of rs175338 genotype on clinical and metabolic variables of patients with newly diagnosed type 2 diabetes in the VNDS. Beta and SE are the multivariate model estimates of the change and its standard deviation, respectively, in the phenotypic trait associated to the presence of one minor allele. Reported p values were obtained after adjusting for age, gender and BMI. *Variables were log-transformed before statistical analysis to approximate a gaussian distribution; the coefficient beta and its S.E. refer to log-transformed values.

|  | | **rs175338** | | | **Additive model** | | |
| --- | --- | --- | --- | --- | --- | --- | --- |
| **Phenotype** | **N** | **GG** | **GA** | **AA** | **BETA** | **SE** | ***P* Value** |
| **Minor allele frequency, %** | | **21** | | |  |  |  |
| Number (M/F) | 572 (392/180) | 348 | 204 | 20 |  |  |  |
| Age (yrs) | 572 | 60 [52-66] | 59 [52-66] | 60 [53-64] | 0.06 | 0.75 | 0.94 |
| BMI* (kg/m^2^) | 572 | 29.4 [26.6-32.9] | 28.7 [25.9-32.5] | 28.2 [25.9-35.3] | -0.01* | 0.01* | 0.34 |
| Fasting P-glucose (mmol/l) | 538 | 7.1 [6.2-8] | 7.2 [6.2-7.9] | 6.7 [6.2-7.6] | -0.15 | 0.14 | 0.27 |
| 2hr P-glucose (mmol/l) | 527 | 13.1 [10.4-16.2] | 13.7 [10.9-16.3] | 10.9 [9.1-14.2] | -0.23 | 0.32 | 0.48 |
| Fasting insulin* (mU/l) | 469 | 11.6 [7.5-17.3] | 10.8 [7.4-14.6] | 12.6 [9.9-17.9] | 0.008* | 0.04* | 0.86 |
| 2hr insulin* (mU/l) | 475 | 66.9 [37.9-100] | 58.9 [38.3-91.1] | 77.8 [49.6-112] | 0.02* | 0.06* | 0.71 |
| Insulinogenic Index (mU/mmol)* | 427 | 4.1 [2.4-7.4] | 3.4 [1.7-6.1] | 6.3 [4.3-11.6] | -0.06* | 0.08* | 0.41 |
| CIR_120’_ *(mUxL/mmol^2^) | 474 | 0.5 [0.2-1.3] | 0.5 [0.2-0.9] | 1 [0.5-1.5] | 0.07* | 0.09* | 0.48 |
| HbA1c | 548 | 6.6 [6.1-7.4] | 6.8 [6.3-7.6] | 6.4 [6-6.8] | 0.04 | 0.10 | 0.66 |
| Triglycerides* (mmol/l) | 554 | 1.5 [1.1-2] | 1 [0.9-2] | 1.5 [1-2.1] | -0.04* | 0.04* | 0.31 |
| HDL-cholesterol* (mmol/l) | 547 | 1.1 [1-1.3] | 1.2 [1-1.4] | 1.2 [1-1.4] | 0.05* | 0.02* | **0.006** |
| Cholesterol (mmol/l) | 554 | 4.9 [4.2-5.6] | 5 [4.3-5.7] | 5.3 [4.8-5.9] | 0.09 | 0.08 | 0.24 |
| SBP (mmHg) | 554 | 135 [120-150] | 136 [120-150] | 137 [124-145] | 0.22 | 1.32 | 0.87 |
| DBP (mmHg) | 554 | 80 [80-90] | 80 [80-90] | 89 [80-90] | 0.40 | 0.71 | 0.60 |
| Basal insulin secretion* | 468 | 136.7 [98.4-181.2] | 135.9 [98.4-169.4] | 135.6 [115.3-162.8] | 0.02* | 0.03* | 0.58 |
| Insulin clearance* ml/min/m^2^ BSA | 518 | 701 [539-828] | 660 [552-797] | 707 [544-794] | -0.04* | 0.03* | 0.14 |
| Insulin Sensitivity*  (µmol/min/m^2^ BSA) | 553 | 587 [368-865] | 614 [408-848] | 664 [313-755] | -0.01* | 0.04* | 0.75 |

Table S10. Influence of rs3753737 genotype on clinical and metabolic variables of patients with newly diagnosed type 2 diabetes in the VNDS. Beta and SE are the multivariate model estimates of the change and its standard deviation, respectively, in the phenotypic trait associated to the presence of one minor allele. Reported p values were obtained after adjusting for age, gender and BMI. *Variables were log-transformed before statistical analysis to approximate a gaussian distribution; the coefficient beta and its S.E. refer to log-transformed values.

|  | | **rs3753737** | | | **Additive model** | | |
| --- | --- | --- | --- | --- | --- | --- | --- |
| **Phenotype** | **N** | **AA** | **AG** | **GG** | **BETA** | **SE** | ***P* Value** |
| **Minor allele frequency, %** | | **27** | | |  |  |  |
| Number (M/F) | 579 (396/183) | 303 | 234 | 42 |  |  |  |
| Age (yrs) | 579 | 60 [53-65] | 59 [51-66] | 59 [50-65] | -0.62 | 0.67 | 0.36 |
| BMI* (kg/m^2^) | 579 | 29.7 [26.5-32.9] | 28.7 [26.7-32.8] | 29.1 [25.7-32.7] | 0.007* | 0.011* | 0.51 |
| Fasting P-glucose (mmol/l) | 544 | 7.3 [6.2-8.2] | 7 [6.2-7.8] | 7 [6-7.8] | -0.19 | 0.13 | 0.13 |
| 2hr P-glucose (mmol/l) | 532 | 13.3 [10.8-16.2] | 12.9 [10.5-16.2] | 12.5 [10.5-16.2] | -0.19 | 0.29 | 0.51 |
| Fasting insulin* (mU/l) | 474 | 11.4 [7.6-16] | 11.3 [7.5-17] | 11.8 [7.5-16.3] | 0.07* | 0.04* | 0.08 |
| 2hr insulin* (mU/l) | 477 | 62.3 [40.1-98.2] | 61.7 [32.3-99.5] | 75.4 [38.2-100] | 0.06* | 0.05* | 0.24 |
| Insulinogenic Index (mU/mmol)* | 430 | 4.3 [2.2-7.1] | 3.7 [2-6.9] | 3.6 [2.3-5.5] | 0.009* | 0.07* | 0.90 |
| CIR_120’_ *(mUxL/mmol^2^) | 476 | 0.5 [0.2-1.1] | 0.5 [0.2-1.4] | 0.5 [0.3-1] | 0.07* | 0.09* | 0.44 |
| HbA1c | 555 | 6.6 [6.2-7.5] | 6.6 [6.1-7.3] | 6.6 [6.3-7.5] | -0.09 | 0.09 | 0.33 |
| Triglycerides* (mmol/l) | 561 | 1.4 [1.1-2] | 1.4 [1-2] | 1.6 [1.1-2.8] | 0.02* | 0.03* | 0.66 |
| HDL-cholesterol* (mmol/l) | 554 | 1.1 [1-1.3] | 1.1 [1-1.4] | 1.1 [0.9-1.3] | 0.001* | 0.02* | 0.95 |
| Cholesterol (mmol/l) | 561 | 5.1 [4.4-5.8] | 4.8 [4.2-5.5] | 4.8 [4.2-5.5] | -0.14 | 0.07 | **0.038** |
| SBP (mmHg) | 560 | 135 [125-150] | 133 [120-150] | 140 [130-150] | 1.60 | 1.18 | 0.18 |
| DBP (mmHg) | 560 | 84 [80-90] | 81 [80-90] | 86 [80-90] | 0.74 | 0.63 | 0.24 |
| Basal insulin secretion* | 472 | 136.8 [102.6-174.7] | 131.1 [97-175.1] | 156.2 [99.3-181.5] | 0.004* | 0.03* | 0.89 |
| Insulin clearance* ml/min/m^2^ BSA | 524 | 699 [543-811] | 675 [533-815] | 693 [584-822] | -0.002* | 0.02* | 0.93 |
| Insulin Sensitivity*  (µmol/min/m^2^ BSA) | 560 | 604 [386-878] | 607 [383-826] | 482 [318-948] | -0.04* | 0.04* | 0.37 |

Table S11. Influence of rs225338 genotype on clinical and metabolic variables of patients with newly diagnosed type 2 diabetes in the VNDS. Beta and SE are the multivariate model estimates of the change and its standard deviation, respectively, in the phenotypic trait associated to the presence of one minor allele. Reported p values were obtained after adjusting for age, gender and BMI. *Variables were log-transformed before statistical analysis to approximate a gaussian distribution; the coefficient beta and its S.E. refer to log-transformed values.

|  | | **rs2253388** | | | **Additive model** | | |
| --- | --- | --- | --- | --- | --- | --- | --- |
| **Phenotype** | **N** | **GG** | **GA** | **AA** | **BETA** | **SE** | ***P* Value** |
| **Minor allele frequency, %** | | **29** | | |  |  |  |
| Number (M/F) | 581 (403/178) | 292 | 244 | 45 |  |  |  |
| Age (yrs) | 581 | 59 [52-64] | 60 [51-67] | 59 [55-66] | 0.63 | 0.70 | 0.34 |
| BMI* (kg/m^2^) | 581 | 29.4 [26.8-32.9] | 29 [26.2-32.8] | 29.7 [25.7-33.4] | -0.01* | 0.01* | 0.51 |
| Fasting P-glucose (mmol/l) | 546 | 7.2 [6.3-8] | 7.1 [6.1-8.3] | 7.2 [6.3-7.6] | -0.01 | 0.13 | 0.96 |
| 2hr P-glucose (mmol/l) | 534 | 13.1 [10.8-16.2] | 13.3 [10.4-16.1] | 13.5 [10.6-16.8] | 0.03 | 0.29 | 0.92 |
| Fasting insulin* (mU/l) | 474 | 11.4 [7.5-16.2] | 10.7 [7.2-16.1] | 13.1 [9.7-16.8] | 0.02* | 0.04* | 0.63 |
| 2hr insulin* (mU/l) | 482 | 62.6 [37.8-100] | 61 [35-94.3] | 75.4 [42.5-108.9] | 0.03* | 0.05* | 0.62 |
| Insulinogenic Index (mU/mmol)* | 432 | 3.9 [2-7] | 4.1 [2.1-7.1] | 3.6 [2.4-5.7] | 0.01* | 0.07* | 0.93 |
| CIR_120’_ *(mUxL/mmol^2^) | 481 | 0.5 [0.2-1.1] | 0.5 [0.2-1.3] | 0.6 [0.2-1.2] | 0.01* | 0.08* | 0.87 |
| HbA1c | 557 | 6.6 [6.1-7.4] | 6.8 [6.2-7.6] | 6.7 [6.1-7.3] | 0.10 | 0.09 | 0.26 |
| Triglycerides* (mmol/l) | 563 | 1.4 [1.1-2.1] | 1.4 [1-2] | 1.3 [1-1.9] | -0.04* | 0.03 | 0.27 |
| HDL-cholesterol* (mmol/l) | 556 | 1.1 [1-1.3] | 1.2 [1-1.4] | 1.2 [1-1.4] | 0.04* | 0.02* | **0.013** |
| Cholesterol (mmol/l) | 563 | 5 [4.3-5.7] | 4.9 [4.3-5.6] | 4.9 [4.2-5.8] | -0.04 | 0.07 | 0.52 |
| SBP (mmHg) | 562 | 131 [120-150] | 140 [128-150] | 135 [120-140] | 0.66 | 1.17 | 0.57 |
| DBP (mmHg) | 562 | 81 [80-90] | 82 [80-90] | 83 [77-90] | -0.35 | 0.62 | 0.58 |
| Basal insulin secretion* | 475 | 136.9 [100.1-176.6] | 132.4 [97.7-167] | 138.7 [100.7-191.1] | -0.007* | 0.03* | 0.79 |
| Insulin clearance* ml/min/m^2^ BSA | 526 | 705 [563-816] | 678 [515-807] | 717 [581-843] | 0.001* | 0.02* | 0.95 |
| Insulin Sensitivity*  (µmol/min/m^2^ BSA) | 562 | 617 [376-857] | 588 [375-865] | 451 [352-710] | -0.08* | 0.04* | **0.046** |

Table S12. Influence of rs4652679 genotype on clinical and metabolic variables of patients with newly diagnosed type 2 diabetes in the VNDS. Beta and SE are the multivariate model estimates of the change and its standard deviation, respectively, in the phenotypic trait associated to the presence of one minor allele. Reported p values were obtained after adjusting for age, gender and BMI. *Variables were log-transformed before statistical analysis to approximate a gaussian distribution; the coefficient beta and its S.E. refer to log-transformed values.

**^§^** For these analysis basal insulin secretion and insulin clearance have been used as additional covariates.

|  | | **rs4652679** | | | **Additive model** | | |
| --- | --- | --- | --- | --- | --- | --- | --- |
| **Phenotype** | **N** | **GG** | **GA** | **AA** | **BETA** | **SE** | ***P* Value** |
| **Minor allele frequency, %** | | **27** | | |  |  |  |
| Number (M/F) | 584 (402/182) | 311 | 234 | 39 |  |  |  |
| Age (yrs) | 584 | 59 [52-65] | 61 [53-67] | 57 [50-64] | 0.50 | 0.68 | 0.46 |
| BMI* (kg/m^2^) | 584 | 29.1 [26.2-32.5] | 29.4 [26.8-32.9] | 29.6 [25.9-33.3] | 0.02* | 0.01* | 0.18 |
| Fasting P-glucose (mmol/l) | 549 | 7.2 [6.2-8.2] | 7 [6.2-7.8] | 6.9 [6.3-7.6] | -0.21 | 0.13 | 0.11 |
| 2hr P-glucose (mmol/l) | 538 | 13.6 [10.8-16.3] | 12.9 [10.3-15.9] | 12.5 [10.1-16.2] | -0.37 | 0.29 | 0.20 |
| Fasting insulin* (mU/l) | 480 | 9.9 [6.7-15.8] | 12.2 [8.5-16] | 13 [9.9-17.3] | 0.09*  0.05***^§^** | 0.04*  0.03***^§^** | **0.02**  0.1**^§^** |
| 2hr insulin* (mU/l) | 485 | 58.4 [33.1-86.6] | 67.2 [44.1-111.5] | 70 [45.8-119] | 0.15*  0.06***^§^** | 0.05*  0.05***^§^** | **0.004**  0.2**^§^** |
| Insulinogenic Index (mU/mmol)* | 437 | 3.7 [2-6.3] | 4.5 [2.2-7.4] | 3.6 [2.4-5.7] | 0.06 | 0.07 | 0.42 |
| CIR_120’_ *(mUxL/mmol^2^) | 484 | 0.4 [0.2-1.1] | 0.6 [0.3-1.4] | 0.7 [0.3-1] | 0.19*  0.13***^§^** | 0.08*  0.09***^§^** | **0.027**  0.1**^§^** |
| HbA1c | 560 | 6.6 [6.1-7.7] | 6.6 [6.2-7.3] | 6.7 [6.2-7.2] | -0.07 | 0.09 | 0.41 |
| Triglycerides* (mmol/l) | 566 | 1.4 [1-2.1] | 1.4 [1-2] | 1.6 [1.1-2] | -0.02* | 0.03* | 0.58 |
| HDL-cholesterol* (mmol/l) | 559 | 1.1 [1-1.3] | 1.1 [1-1.3] | 1.1 [0.9-1.4] | 0.01* | 0.02* | 0.72 |
| Cholesterol (mmol/l) | 566 | 5 [4.4-5.8] | 4.8 [4.2-5.5] | 4.9 [4-5.3] | -0.17 | 0.07 | **0.015** |
| SBP (mmHg) | 565 | 135 [120-150] | 140 [125-150] | 130 [124-150] | -0.56 | 1.19 | 0.64 |
| DBP (mmHg) | 565 | 80 [80-90] | 84 [80-90] | 80 [80-90] | -0.38 | 0.63 | 0.55 |
| Basal insulin secretion rate* | 479 | 129.3 [95.9-171] | 139.3 [107.8-178.6] | 144.3 [107.7-189.1] | 0.06* | 0.03* | **0.031** |
| Insulin clearance* ml/min/m^2^ BSA | 530 | 710 [557-844] | 674 [528-798] | 687 [517-745] | -0.07* | 0.02* | **0.003** |
| Insulin Sensitivity*  (µmol/min/m^2^ BSA) | 565 | 614 [385-874] | 582 [358-819] | 670 [434-784] | 0.01* | 0.04* | 0.99 |

**Table S13**. Number of patients with newly diagnosed type 2 diabetes of the VNDS in each cell of the *CACNA1E* score. We considered two levels for rs2184945 (TT and AA/AT) and 3 levels for rs3905011 (AA, AG and GG) and we scored them from 0 to 1 or to 2 respectively. The *CACNA1E* score could range from a minimum of 0 (double genotype rs2184945TT – rs3905011AA) to a maximum of 3 (a rs2184945AA/AT – rs3905011GG genotype).

| Genotype Score | rs2184549  TT=0  AA/AT=1 | rs3905011  AA=0  AG=1  GG=2 | N |
| --- | --- | --- | --- |
| 0 | TT | AA | 24 |
| 1 | TT | AG | 101 |
|  | AA/AT | AA |  |
| 2 | AA/AT | AG | 233 |
|  | TT | GG |  |
| 3 | AA/AT | GG | 151 |

**Online figure legends.**

Fig. S1a. Effects of rs2184549 on glucose stimulated insulin secretion rate (proportional control of beta cell function) during the OGTT. The A allele of rs2184549 , according to a dominant model, IS associated with a statistically significant reduction in beta cell function.

Fig. S1b. Effects of rs3905011 on glucose stimulated insulin secretion rate (proportional control of beta cell function) during the OGTT. The G allele of rs3905011 is associated with a statistically significant reduction in beta cell function.

**References**

1. Monauni T, Zenti MG, Cretti A, Daniels MC, Targher G, et al. (2000) Effects of glucosamine infusion on insulin secretion and insulin action in humans. Diabetes 49: 926-935.

2. Bonadonna RC, del Prato S, Bonora E, Gulli G, Solini A, et al. (1993) Effects of physiological hyperinsulinemia on the intracellular metabolic partition of plasma glucose. Am J Physiol 265: E943-953.

3. Cali AM, Bonadonna RC, Trombetta M, Weiss R, Caprio S (2008) Metabolic abnormalities underlying the different prediabetic phenotypes in obese adolescents. J Clin Endocrinol Metab 93: 1767-1773.

4. Weiss R, Caprio S, Trombetta M, Taksali SE, Tamborlane WV, et al. (2005) Beta-cell function across the spectrum of glucose tolerance in obese youth. Diabetes 54: 1735-1743.

5. Cobelli C, Toffolo GM, Dalla Man C, Campioni M, Denti P, et al. (2007) Assessment of beta-cell function in humans, simultaneously with insulin sensitivity and hepatic extraction, from intravenous and oral glucose tests. Am J Physiol Endocrinol Metab 293: E1-E15.

6. Mari A, Camastra S, Toschi E, Giancaterini A, Gastaldelli A, et al. (2001) A model for glucose control of insulin secretion during 24 h of free living. Diabetes 50 Suppl 1: S164-168.

7. Van Cauter E, Mestrez F, Sturis J, Polonsky KS (1992) Estimation of insulin secretion rates from C-peptide levels. Comparison of individual and standard kinetic parameters for C-peptide clearance. Diabetes 41: 368-377.

8. Toschi E, Camastra S, Sironi AM, Masoni A, Gastaldelli A, et al. (2002) Effect of acute hyperglycemia on insulin secretion in humans. Diabetes 51 Suppl 1: S130-133.

9. Foster DM, Boston RC, Jacquez JA, Zech L (1989) A resource facility for kinetic analysis: modeling using the SAAM computer programs. Health Phys 57 Suppl 1: 457-466.
