# Supplementary figures and images for: CACNA1E Variants Affect Beta Cell Function in Patients with Newly Diagnosed Type 2 Diabetes. The Verona Newly Diagnosed Type 2 Diabetes Study (VNDS) 3
Source: PLoS One. 2012 Mar 9;7(3):e32755. doi: 10.1371/journal.pone.0032755 (PMC3302892; doi:10.1371/journal.pone.0032755)

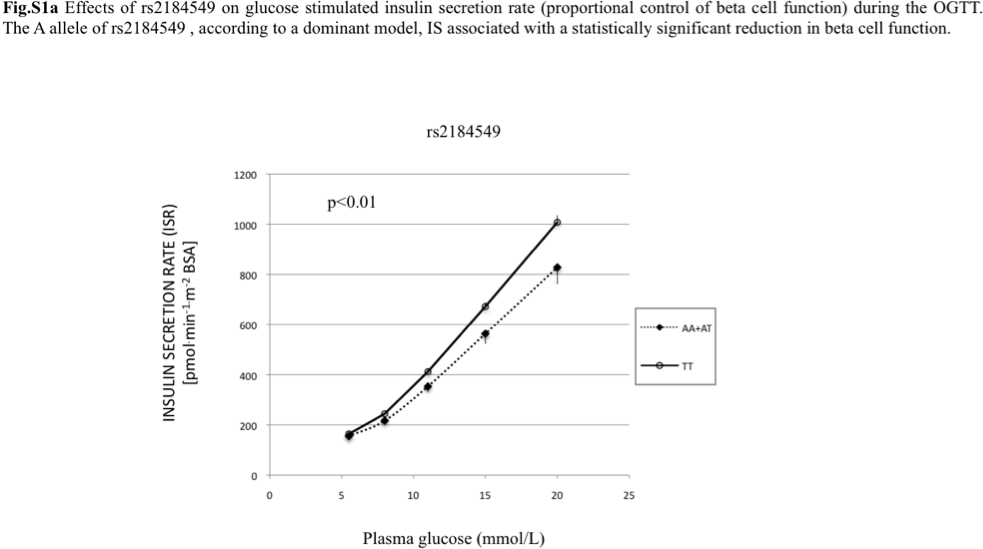


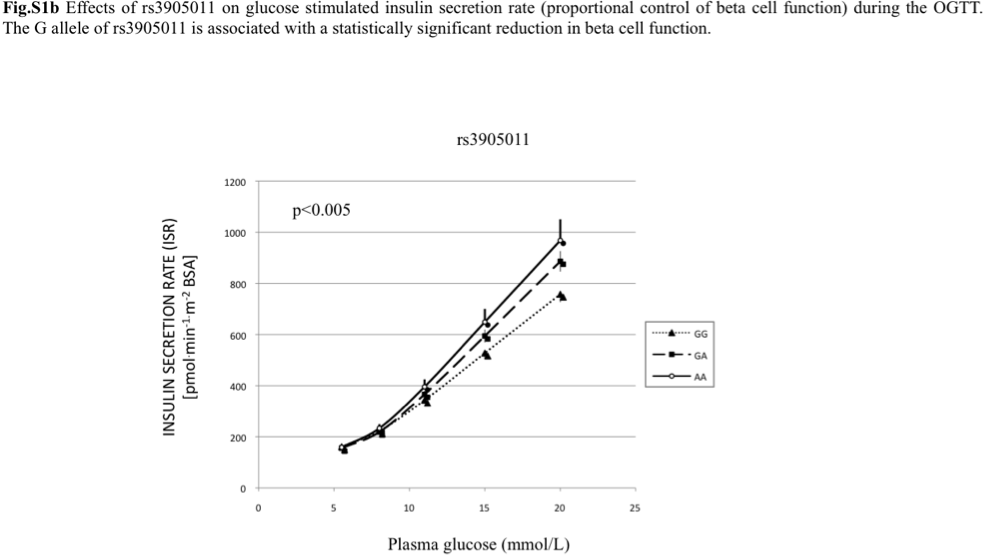

Supplement: Supporting Information S2 — Includes supplemental figures S1a and S1b. (DOC) [file pone.0032755.s002.doc]
